# Prophylactic antibiotics used in patients of hepatobiliary surgery

**Published:** 2013

**Authors:** Jianjun Ren, Lidao Bao, Jianxiang Niu, Yi Wang, Xianhua Ren

**Affiliations:** 1 Jianjun Ren, Department of General Surgery, Affiliated Hospital of Inner Mongolia Medical University, Hohhot-010059, P. R. China.; 2 Lidao Bao, Department of Pharmacy, Affiliated Hospital of Inner Mongolia Medical University, Hohhot-010059, P. R. China.; 3 Jianxiang Niu, Department of General Surgery, Affiliated Hospital of Inner Mongolia Medical University, Hohhot-010059, P. R. China.; 4 Yi Wang, Department of Pharmacy, Affiliated Hospital of Inner Mongolia Medical University, Hohhot-010059, P. R. China.; 5 Xianhua Ren, Affiliated Hospital of Inner Mongolia Medical University, Hohhot-010059, P. R. China.

**Keywords:** Prophylactic, Antibiotics, Hepatobiliary, Surgical site infection

## Abstract

***Objective:***To clarify the use of antibiotics in our hospital and to guide the prophylactic use in future hepatobiliary surgical procedures.

***Methodology:*** A retrospective review of patients who underwent hepatobiliary surgery from January 2011 to June 2011 was included. Data were collected, and surgical site infection (SSI) was defined by the criteria of Center for Disease Control and Prevention. Patients were prescribed antibiotics for the clinical diagnosis of hepatobiliary system diseases.

***Results:*** 1564 patients were identified, in which 784 patients (50.13%) did not receive preoperative antibiotic prophylaxis. Of these 355 patients with 784 surgical sites received either preoperative or both preoperative and postoperative antibiotic prophylaxis. The SSI rate of the patients who received prophylaxis alone (2.56%, 20 of 780 sites) was not statistically higher than that of the patients who have not received prophylaxis (2.68%, 21 of 784 sites), and the two groups were not statistically correlated (P=0.77).

***Conclusion:*** The number of the patients who developed SSI was relatively low, and no reduction in the SSI rate was observed among the patients who have received antibiotic prophylaxis.

## INTRODUCTION

In the clean operative field, antibiotics effectively prevent surgical site infection (SSI). Prophylactic antibiotics are able to not only reduce the SSI, but also decrease the incidence of drug resistance.^1^^,^^2^ However, prophylactic antibiotics are not required in all the surgery. Besides, SSI refers to the infection of surgical incision or deep organ/space following the surgery, including incision infection, abscess peritonitis, etc.^3^ SSI accounts for 15% of the hospital acquired infection and 35 to 40% of the infection in the department of surgery.^4^ Recently, surgical complications result in significant cost to the individual, community and healthcare system.^5^ It has been reported that the incidence of wound infection was about 1% in aseptic operative procedure,^6^ 2 to 5% of the patients undergoing the clean extra abdominal operations and up to 20% of them undergoing intra abdominal operations would develop into SSI.^7^ Currently, the perioperative use of prophylactic antibiotics is commonly used in the hepatobiliary operative procedures.

The efficacy of the antimicrobial prophylaxis for the prevention of SSIs was established in the 1960s and has been demonstrated repeatedly since then.^8^Moreover, surgical site infection (SSI) prophylaxis with one preoperative dose of an intravenously administered antibiotic that was of antistaphylococcal activity before the hepatobiliary surgery has been utilized as the standard of care. Up to a 5-fold increase in the SSI risk was found in the patients with the hepatobiliary operation compared to those who have not undergone hepatobiliary procedures.^9^It has also been reported that the routine postoperative antibiotic administration would positively affect the prevention of SSI after hepatobiliary operations.^10^ Furthermore, specific guidelines outline the indications for antibiotic prophylaxis, based upon operation types and patient characteristics.No prophylaxis was carried out in superficial skin surgery and simple mucosal excisions.

Antibiotic prophylaxis is always indicated in microsurgery, prosthetic surgery, incisional hernias, clean non-prosthetic osteoarticular surgery and contaminated procedures such as oral cavity or genitourinary system. In the clean surgery and rhinoplasty, antibiotic prophylaxis is only indicated when the operation lasts more than 3 hours and/or the American Society of Anesthesiologists (ASA) score is no less than three. The risk of infection can be kept very low with the reported protocol, which would avoid the side effect of indiscriminate use of antibiotics.^11^Furthermore, many surgeons prescribe prophylaxis for all the patients with hepatobiliary surgery to prevent infection. Some studies have described the use of prophylactic antibiotics in hepatobiliary procedures when surgical drains are in place with the assumption that antibiotic prophylaxis would decrease the SSI risks.^12^

Surgical drains are commonly removed 5–7 days later when the output is 30 ml per 24 h. However, drains would be kept in place for some patients for weeks before meeting the removal criteria. The study herein retrospectively investigated the effect of prophylactic antibiotics on the perioperative period of hepatobiliary operations for SSI rates in a single institution cohort.^2^

## METHODOLOGY

***Patients:***The study was approved by the Ethics Committees of Affiliated Hospital of Inner Mongolia Medical University. In the present study, a total of 1564 patients with a mean age of 53±9 years old with hepatobiliary operative procedures from January to June in 2011 were selected and retrospectively reviewed, which presented the current situation of prophylactic use of antibiotics in the hepatobiliary operative procedures and provided evidence for the further development of principles for prophylactic antibiotics in the hepatobiliary operative procedures.Detailed operative procedures included left liver resection,hepatoduodenal ligament skeletonization,T tube drainage,resection of post-peritoneal neurinoma, cholecystectomy, laparotomy,splenectomy, and double inguinal hernioplasty, etc.The characteristics and information of the patients toward antibiotics were recorded and reviewed.

All the patients were divided into two groups depending on whether they had been administered with antibiotics to prevent incision infections: The prophylaxis group received at least one dose of antibiotic in the course of treatment and the other group did not receive antibiotic prophylaxis.The number of cases in the two groups almost equaled because the antibiotic prophylaxis remained unclear in our department before this study and thus the patients were randomly administered.

***Characteristics of the patients*** *:* Name, gender, age, body weight, case number and hospitalization days, diagnosis, surgery name, date for surgery, the time of surgery initiation and completion were recorded.

***Information of Antibiotics:***No prophylaxis was carried out in superficial skin surgery and simple mucosal excisions. Antibiotic prophylaxis is always indicated in microsurgery, prosthetic surgery, incisional hernias, clean non-prosthetic osteoarticular surgery and contaminated procedures such as oral cavity or genitourinary system.Name, formulation, dose, usage, total dose, date for antibiotic initiation and completion and durations of pre-operative and post-operative antibiotics was noted.

***Dates of SSI:*** SSI was defined by means of Centers for Disease Control and Prevention criteria: 1) purulent drainage; 2) positive aseptically obtained culture; 3) peri-incisional erythema on incision opened by the surgeon; and 4) physician diagnosis of infection, which was pre-dominantly a diagnosis of cellulites. Dates of SSI were collected with rates calculated for a 30-day postoperative period and Fisher’s exact test was employed to compare SSI rates of the patients with and without receiving postoperative antibiotic prophylaxis. Logistic regressions were then used to assess the effect of antibiotic prophylaxis and adjust for potential confounding variables as well.^13^

**Table-I:** General information of the two groups

*Operation*	*Gender*		*Age, y* *mean ±SD*	*Average length of stay/day*	*Infection risk factors*			*Fervescence in perioperative period*
	*Male*	*Female*			*>70 years*	Malignancy	*Diabetes mellitus*	
With prophylactic antibiotics	366	414	52.5±11.7	18.2	11	9	1	5
Without prophylactic antibiotics	375	409	54.2±8.5	17.9	7	1	3	
Sum total	741	823			18	10	4	5

**Table-II:** SSI rates of the groups in the presence and absence ofpostoperative antibiotic prophylaxis

	*Cases*	*Surgical site infection rates* *by hepatobiliary surgery*
All surgical drains	1564	41/1564(2.62%)
With Prophylactic Antibiotics	780(49.87%)	20/780(2.56%)
Without Prophylactic Antibiotics	784(50.13%)	21/784(2.68%)
P value		0.77

The two groups did not differ significantly

**Table-III:** Antibiotics used for postoperative prophylaxis

*General Name*	*Frequency*	*Constituent ratio (%)*
Cefodizime sodium injection	201	25.77
Cefmenoxime injection	174	22.31
Cefoperazone / tazobactam sodium injection	112	14.36
Cefpiramide sodium injection	75	9.62
Meropenem injection	71	9.10
Cefamandole Nafate for Injection	56	7.18
Flucloxacillin sodium injection	39	5.00
Sulbenicillin Sodium injection	24	3.08
Levofloxacin injection	22	2.82
Latamoxef sodium injection	6	0.77
Sum total	780	100.00

Cefodizime sodium injection was most frequently used, and the top three antibiotics accounted for 62.44% of all administration

## RESULTS

In this retrospective single-institution study, no differences of the SSI rate of the patients having received single-dose preoperative antibiotics before hepatobiliary surgery were discovered compared to those having received both preoperative and postoperative antibiotic prophylaxis.1564 patients who have undergone hepatobiliary operations were identified during the study process. 784 patients (50.13%) did not receive preoperative antibiotic prophylaxis, and 355 patients with 780 surgical sites received either preoperative or both preoperative and postoperative antibiotic prophylaxis. When the analysis of the procedures with drains was restricted, the two groups were similar differing in only the average age. In the prophylaxis group, the medium length of stay was 18.2 days (range, 5–44 days), whereas the time of the group without prophylactic antibiotic was 17.9 days (range, 3–41 days). When the analysis was restricted to the patients who have received hepatobiliary surgery, the SSI rate of the patients who received prophylaxis alone (2.56%, 20 of 780 sites) was not statistically higher than that of the patients who did not receive prophylaxis (2.68%, 21 of 784 sites). Besides, adjustment for possible indications for antibiotic prophylaxis (age, malignancy, diabetes mellitus, and fervescence in perioperative period)^14^^,^^15^ did not reveal a statistically significant difference (P=0.77) (Table-I).

The SSI rate of 632 procedures involved in the vascular intervention operation overall was 2.62% (41 of 1564 surgical sites). In general, the SSI rates seemed higher in the patients undergoing more extensive procedures accompanied by the drain placement, but these differences were not statistically significant except for the differences between the patients without drains and those with splenectomy. The presence of purulent drainage and the positive culture by the surgeon constituted the minority of SSI (29.27%, 12 of 41), Besides, the SSI rates did not differ statistically (P=0.77) in the group in the presence (2.56%; 95% confidence interval [95% CI], 4.5–14.2; 20 of 780 surgical sites) and absence of (2.68%; 95% confidence interval [95% CI], 4.9–13.8; 21 of 784 surgical sites) postoperative antibiotic prophylaxis (Table-II).In this retrospective single-institution study, no differences of the SSI rate of the patients having received single-dose preoperative antibiotics before hepatobiliary surgery were discovered compared to those having received both preoperative and postoperative antibiotic prophylaxis.

870 patients (49.84%) with 1564 surgical sites were provided with both pre- and postoperative prophylactic antibiotics. Indications for postoperative prophylaxis (n=63) included neoadjuvant chemotherapy (n=17), diabetes mellitus (n=26), tobacco use (n=49) and corticosteroid dependence (n=25) initiated before the surgery for other active infectious diseases and continued through the postoperative period (n=30) and surgeon’s discretion (n=158).Cefazolin was regularly utilized for preoperative prophylaxis unless the patient had a documented allergy. The antibiotics used for postoperative prophylaxis consisted of cefodizime sodium injection (25.77%), cefmenoxime injection (22.31%), cefoperazone / tazobactam sodium injection (14.36%), and others (37.56%) (Table-III).

## DISCUSSION

SSIs occur more frequently after hepatobiliary surgery than that expected for the aseptic cases.^16^ Whether antibiotic prophylaxis in orthognathic surgery could effectively reduce the postoperative infection rate has been investigated by Tan et al. Five randomized clinical trials were included in the final review process: four of them compared the period of the prophylactic antibiotic usage, and the other one compared the infection prevention performance of different types of antibiotics with that of the placebo. Although a significantly higher infection rate was found in the placebo group, no significant differences could be found related to the infection prevention between the short-term and long-term antibiotic regimen.^17^

A multivariate analysis of SSI risk factors in the hepatobiliary operations revealed that blood loss was still the major concern. Besides, age, malignancy, diabetes mellitus and fervescence in the perioperative period were also the risk factors.^18^ Moreover, it is likely that patients were given postoperative prophylaxis by the surgeons due to the higher risk of SSI, which would result in potentially higher risk for SSI of the treatment group. Meanwhile, almost all the tested patients^19^ who underwent hepatobiliary surgery also received postoperative antibiotics, the characteristics of the patients having received antibiotics was almost exactly the same as those of the group having not received prophylaxis.

The SSI rates were higher than expected after the aseptic hepatobiliary procedures, but it is lack of evidence for the benefit of prophylactic antibiotics, and the infection risks increased due to the resistant organisms.^20^ The trial investigated the advantages of antibiotic prophylaxis in reducing the SSI rate after the hepatobiliary surgery, which assists to identify the patients for whom this treatment is implemented.

In summary, no statistically significant SSI reduction occurred among the patients who received preoperative and postoperative antibiotic prophylaxis compared to those with preoperative antibiotic prophylaxis alone. However, the potential adverse events associated with the antibiotic use ought to be recognized, and further evaluation of the practice is still needed .
